# Assessing the usefulness of a novel MRI-based breast density estimation algorithm in a cohort of women at high genetic risk of breast cancer: the UK MARIBS study

**DOI:** 10.1186/bcr2447

**Published:** 2009-11-11

**Authors:** Deborah J Thompson, Martin O Leach, Gek Kwan-Lim, Simon A Gayther, Susan J Ramus, Iqbal Warsi, Fiona Lennard, Michael Khazen, Emilie Bryant, Sadie Reed, Caroline RM Boggis, D Gareth Evans, Rosalind A Eeles, Douglas F Easton, Ruth ML Warren

**Affiliations:** 1 Cancer Research UK Genetic Epidemiology Unit, Department of Public Health and Primary Care, University of Cambridge, Worts Causeway, Cambridge, CB1 8RN, UK; 2 Institute of Cancer Research and Royal Marsden NHS Foundation Trust, Downs Road, Sutton, Surrey, SM2 5PT, UK; 3 Gynaecological Cancer Research Centre, UCL EGA Institute for Women's Health, University College London, Gower Street, London, WC1E 6BT, UK; 4 Department of Radiology, University of Cambridge, Addenbrookes Hospital NHS Foundation Trust, Hills Road, Cambridge, CB2 0QQ, UK; 5 Breast Screening Unit, Nightingale Centre & Genesis Prevention Centre, Wythenshawe Hospital, Southmoor Road, Manchester M23 9LT, UK; 6 Academic Unit of Medical Genetics and Regional Genetics Service, St Mary's Hospital, Hathersage Road, Manchester, M13 0JH, UK; 7 See Additional data file 1 for full authorship list

## Abstract

**Introduction:**

Mammographic breast density is one of the strongest known risk factors for breast cancer. We present a novel technique for estimating breast density based on 3D T1-weighted Magnetic Resonance Imaging (MRI) and evaluate its performance, including for breast cancer risk prediction, relative to two standard mammographic density-estimation methods.

**Methods:**

The analyses were based on MRI (n = 655) and mammography (n = 607) images obtained in the course of the UK multicentre magnetic resonance imaging breast screening (MARIBS) study of asymptomatic women aged 31 to 49 years who were at high genetic risk of breast cancer. The MRI percent and absolute dense volumes were estimated using our novel algorithm (MRIBview) while mammographic percent and absolute dense area were estimated using the Cumulus thresholding algorithm and also using a 21-point Visual Assessment scale for one medio-lateral oblique image per woman. We assessed the relationships of the MRI and mammographic measures to one another, to standard anthropometric and hormonal factors, to BRCA1/2 genetic status, and to breast cancer risk (60 cases) using linear and Poisson regression.

**Results:**

MRI percent dense volume is well correlated with mammographic percent dense area (R = 0.76) but overall gives estimates 8.1 percentage points lower (*P *< 0.0001). Both show strong associations with established anthropometric and hormonal factors. Mammographic percent dense area, and to a lesser extent MRI percent dense volume were lower in BRCA1 carriers (*P *= 0.001, *P *= 0.010 respectively) but there was no association with BRCA2 carrier status. The study was underpowered to detect expected associations between percent density and breast cancer, but women with absolute MRI dense volume in the upper half of the distribution had double the risk of those in the lower half (*P *= 0.009).

**Conclusions:**

The MRIBview estimates of volumetric breast density are highly correlated with mammographic dense area but are not equivalent measures; the MRI absolute dense volume shows potential as a predictor of breast cancer risk that merits further investigation.

## Introduction

Mammographic breast density is usually defined as the proportion of a mammographic image occupied by radiodense tissue (largely stromal and epithelial tissues, appearing as white regions) as opposed to nondense, fatty tissue (the darker regions of the image). Thus defined, a high degree of mammographic density is one of the strongest known risk factors for breast cancer. A recent meta-analysis of 42 studies reported an almost five-fold increase in risk between the most dense and least dense groups of women [1]. The association between high density and risk is also believed to be present in women who are already at an elevated risk of breast cancer as a result of carrying a mutation in the *BRCA1 *or *BRCA2 *genes [2]. While continuous, quantitative measures of breast density provide more accurate predictions of breast cancer risk than the older qualitative measures (e.g. BIRADS or Wolfe scoring systems) [1], it is still not clear that optimal relevant information can be obtained from a two-dimensional mammographic image.

Using data from the UK MARIBS (magnetic resonance imaging in breast screening) study, we have developed a novel technique based on three-dimensional T1-weighted Magnetic Resonance Imaging (MRI) for obtaining estimates of the absolute and proportional volumes of the breast occupied by dense tissue [3]. The MRI density differs from that obtained from mammography in that it is based on a three-dimensional image of the breast and differentiates between *dense *and *non-dense *tissue on the basis of behaviour in response to a magnetic field rather than to x-ray radiation. We considered both the absolute volume occupied by dense material, and the dense volume as a proportion of the entire breast volume.

In the absence of an established biological gold-standard measure of the quantity of dense tissue in the breast, we evaluated the performance of our MRI-based method via direct comparison with two mammography-based methods and by assessing correlations with known anthropometric and hormonal determinants of breast density. We also looked at the relationships between MRI and mammographic breast density measures and *BRCA1*/2 genetic status, and attempted to assess their relative usefulness for breast cancer risk prediction.

The MARIBS study was restricted to carriers of *BRCA1*, *BRCA2 *or *TP53 *mutations, their relatives and other women believed to be at high genetic risk of breast cancer. Hence the results of this study relate to the group of women in whom breast MRI for breast screening is most likely to take place, and therefore for whom MRI-based density estimation is most likely to be possible without the need for additional examinations. In addition it has potential value as a radiation-free density-estimation technique suitable for use from a young age in radiation-susceptible women.

## Materials and methods

### MARIBS study design

The study population was taken from women who participated in the MARIBS study [4,5]., in which 837 asymptomatic women aged 31 to 49 years thought to be at high genetic risk of breast cancer (carriers of *BRCA1*, *BRCA2 *or *TP53 *mutations, their first degree relatives, or untested women with a strong family history of breast and/or ovarian cancer, or a family history suggestive of Li-Fraumeni syndrome; estimated annual risk of breast cancer ≥ 0.9%) consented, of whom 741 attended for MRI and/or X-ray mammography (XRM) in at least one year. All MARIBS participants had given informed consent to a protocol approved by the London Multicentre Research Ethics Committee (reference no. MREC98/2/38) and had specifically consented to their anonymised medical images from the study being used for teaching and research purposes.

### Quantitative mammographic dense area estimation by interactive thresholding

After the close of the study the mammograms were requested from the study centres and from other hospitals which provided routine mammography to study participants (23 hospitals). The recall of mammograms provided a useable image for 607 women (contralateral side only for the 42 women who developed breast cancer; a useable image was only available for one side for a further 31 women). The 607 women included 33 who had consented to participate in the MARIBS study but who had not attended for a study MRI or XRM examination; in these cases the density estimation was based on mammographic images taken outside the context of the MARIBS study. The multi-centre design of the study restricted our ability to trace mammograms from all the women, in part because some centres routinely destroy mammograms after a certain period. Original mammograms retrieved from the centres were digitised using an Array 2905 DICOM ScanPro Plus Laser Film Digitiser Version 1.3E (Array Corp., Hampton, NH, USA) at optical density of 4.7.

The Medial-Lateral Oblique (MLO) images from each breast were used for mammographic density assessment by the two-dimensional interactive method developed by the University of Toronto (CUMULUS V3.1) [6] This method estimates dense breast area and whole breast area from scanned mammograms and hence percentage density. The pectoral muscle is excluded from the image before measuring. The craniocaudal (CC) images were also read for the 481 women for which they were available. RW read all images and also independently second read 158 images for repeatability analyses. All images were read as individual images blinded to all patient information and all other readings.

### Visual Assessment of mammographic dense area

Mammographic density was also assessed visually by an experienced radiologist (CB) for images from 599 women. The digitised MLO images were viewed individually on a standard PC and a percentage estimate of density to 5% was made by the radiologist using a 21-point scale. This method is designed to detect differences smaller than one Boyd category. The craniocaudal (CC) images were also read for the 456 women for which they were available. CB read all the images and also independently second read 93 images for repeatability analyses.

### MRI-based estimation of dense volume

Breast MRI images were obtained for 655 women. The method of density measurement used was devised for this analysis and a pilot study has been published previously [3]. In brief, water-containing tissues were identified by interactive segmentation of tissues anterior to the pectoral muscle on the basis of signal intensity in the pre-contrast T1 weighted images. A coil-uniformity correction based on the proton density image was applied prior to segmentation of water-based and fat-based tissues. Percentage MRI density was calculated as the ratio of the volume occupied by MRI water-containing tissue to the total volume of breast tissue.

Only the contralateral side was considered for the 55 women who developed breast cancer; two further women only had readable images from one side. Wherever possible the density reading was based on the first MRI study conducted in the MARIBS study (n = 645), with the second year study being used for the remaining 10 women. Two readers (EB and SR) were trained to obtain consistent readings using this experimental method. EB read the images for 196 women and SR read those for the remaining 459 women. For repeatability analyses SR independently second read images from 97 of the women originally read by EB (left breasts only used in repeatability analysis to avoid non-independence of the two sides from the same woman).

In summary, the breast density was estimated using both Cumulus and MRI for 513 women, with 142 women having just a MRI density estimate and 94 having just a Cumulus estimate, giving a total of 749 participants. Where more than one mammogram could be traced we used that which was closest in time to the used MRI study; in 423 cases (82.5%) the mammogram was from the same screening round as the MRI image, with a further 42 cases being one year apart (i.e. 90.6% were no more than one year apart).

### Breast cancers in the MARIBS study

Thirty-three screen detected and two interval breast cancers were included in the original report of the MARIBS study in 2005 [5]. An additional four cancers occurred during the study but were excluded from that analysis because they did not have matched MRI and XRM screening episodes. Thirty-eight of the 39 women with breast cancer have density estimates for MRI and XRM (n = 25), for MRI only (n = 12) or for XRM only (n = 1, who had declined an MRI examination), so are eligible for this analysis. Mammograms could not be traced for 12 cases, explaining the discrepancy in numbers.

Information on breast cancers diagnosed after the end of the study was obtained via a follow up questionnaire sent to participants in 2006 and by contacting each of the study centres. Women were also flagged at the Office of National Statistics (ONS) to ascertain subsequent cancer incidence and mortality (having given their written consent). Final follow up including at least 52 months from the last MRI scan was censored at 31 July 2007. We were thus made aware of 28 cancers which emerged since the study closed, of which 22 were eligible for this analysis (13 had MRI and XRM density estimates, 6 had MRI only and 3 had XRM only), giving a total of 60 cases.

This study therefore reflects a combination of prevalent (detected at the time of the screening examination used in the density-estimation, for which density estimates used only the contra-lateral breast) and incident (diagnosed subsequent to the screening examination used in the density estimation, either during a subsequent screening round, or as an interval or follow-up cancer) breast cancers. For the MRI density analysis there were 19 prevalent and 37 incident cancers; for the Cumulus analysis the numbers were 15 and 27 respectively.

### Genetic analyses in the MARIBS population

On the basis of clinical genetic testing independent of this study we were aware of 94 *BRCA1 *and 55 *BRCA2 *mutation carriers and 54 *BRCA1*/2 non-carriers among the 749 women. Fourteen women had undergone clinical genetic testing for *TP53 *mutations (12 positive and 2 negative).

Throughout this report we use the term *non-carrier *to refer to a woman who has tested negative for a specific mutation known to be carried by an affected relative, whereas we describe women as having an *uninformative *test result if no mutations were detected by a general mutation screen. Women whose genetic status was not known at recruitment, including those with an uninformative test result, were invited to provide a blood sample for later anonymous genetic testing. Seven women were anonymously tested for mutations in the *TP53 *gene and 339 were tested for *BRCA1 *and *BRCA2 *mutations by complete gene sequencing to identify coding sequence mutations and by Multiple Ligation-Dependent Probe Amplification (MLPA) to identify large genomic rearrangements and deletions, as previously described [7]. Variants of unknown significance not classed as clinically important by the Breast Cancer Information Core [8] were assumed to be non-deleterious.

Combining clinical test results with the results of our anonymous genotyping we identified 125 *BRCA1 *mutation carriers, 80 *BRCA2 *mutation carriers and 12 carriers of mutations in the *TP53 *gene. One hundred and two women tested negative for a known family mutation (56 *BRCA1*, 41 *BRCA2 *and 5 *TP53*) [Table S1 in Additional data file 2].

The details of the remaining women are given in table S1 in Additional data file 2. Pedigrees were available for 112 of the women without any genetic testing and for 296 of the 303 with an uninformative *BRCA1*/2 test result (either clinical or research). These were used to estimate each woman's probability of carrying a *BRCA1 *or *BRCA2 *mutation given her personal and family history of cancer and mutation testing, based on the Boadicea computer program [9]. The sensitivity parameters for *BRCA1 *and *BRCA2 *mutation screening were set as 70% and 80% respectively. In summary, 70 women had not been tested and had had no testing in their family (median Boadicea carrier probability = 9.0% IQR (inter quartile range) = 1.8 to 23.3%); 42 had not been tested but were related to a tested *BRCA1*/2 mutation carrier (median Boadicea carrier probability = 28.9% IQR = 22.8 to 31.9%) and the remaining 299 had been screened for *BRCA1*/2 mutations (or a relative had) without a deleterious mutation having been detected. Using clinical criteria this last group are considered as *uninformative*, although the thoroughness of the mutation screening suggests that they are unlikely to be missed mutation carriers. This is supported by a median Boadicea probability of not carrying a *BRCA1 *or *BRCA2 *mutation of 98.4% [IQR = 91.9 to 99.6%].

### MARIBS questionnaire information

At the time of recruitment 576 of the 749 women (76.9%) completed a questionnaire containing questions relating to lifestyle risk factors, reproductive and hormonal history and various standard anthropometric measures (Table 1 and Table 2).

**Table 1:** Relationship between measures of breast density and eight continuous variables, by quartile

				Quartile 2	Quartile 3	Quartile 4
	N	P_trend_	P_het_	Coefficient (95% CI)	Coefficient (95% CI)	Coefficient (95% CI)
**MRI % dense volume (log)**						
age	655	< 0.001	< 0.001	-0.02 (-0.14, 0.11)	-0.08 (-0.22, 0.06)	-0.38 (-0.52, -0.25)
weight	519	< 0.001	< 0.001	-0.29 (-0.42, -0.16)	-0.43 (-0.56, -0.29)	-0.83 (-0.97, -0.70)
BMI	517	< 0.001	< 0.001	-0.22 (-0.35, -0.09)	-0.48 (-0.61, -0.35)	-0.86 (-0.99, -0.72)
hip	429	< 0.001	< 0.001	-0.17 (-0.31, -0.03)	-0.29 (-0.45, -0.12)	-0.68 (-0.84, -0.53)
Waist	438	< 0.001	< 0.001	-0.10 (-0.25, 0.06)	-0.29 (-0.43, -0.14)	-0.65 (-0.81, -0.49)
Chest	449	< 0.001	< 0.001	-0.22 (-0.36, -0.09)	-0.37 (-0.52, -0.22)	-0.86 (-1.01, -0.70)
WHR	427	0.006	0.032	-0.09 (-0.26, 0.08)	-0.09 (-0.26, 0.08)	-0.26 (-0.43, -0.09)
Parity	633	0.008	0.052	-0.19 (-0.36, -0.01)	-0.19 (-0.33, -0.05)	-0.22 (-0.39, -0.05)
						
**Cumulus % dense area**						
age	607	0.003	0.004	-1.58 (-5.87, 2.71)	-0.53 (-5.20, 4.14)	-7.66 (-12.2, -3.08)
weight	464	< 0.001	< 0.001	-9.21 (-13.7, -4.72)	-17.2 (-21.7, -12.7)	-26.6 (-31.0, -22.1)
BMI	462	< 0.001	< 0.001	-7.97 (-12.4, -3.56)	-15.7 (-20.1, -11.3)	-27.6 (-32.0, -23.3)
hip	378	< 0.001	< 0.001	-5.59 (-10.4, -0.78)	-10.7 (-16.3, -5.10)	-24.4 (-29.3, -19.6)
Waist	386	< 0.001	< 0.001	-3.56 (-8.99, 1.86)	-11.3 (-16.2, -6.47)	-20.9 (-26.0, -15.8)
Chest	397	< 0.001	< 0.001	-9.73 (-14.2, -5.28)	-13.9 (-18.9, -8.89)	-29.6 (-34.7, -24.6)
WHR	375	0.003	0.005	-6.14 (-11.8, -0.49)	-3.18 (-8.95, 2.59)	-10.1 (-15.9, -4.41)
Parity	596	< 0.001	0.002	-5.70 (-11.2, -0.20)	-5.82 (-10.4, -1.26)	-8.70 (-14.1, -3.27)
						
**Visually Assessed % dense area**						
age	599	0.001	0.004	-1.69 (-6.13, 2.75)	-1.71 (-6.52, 3.10)	-8.34 (-13.1, -3.60)
weight	458	< 0.001	< 0.001	-6.87 (-11.7, -2.05)	-14.1 (-18.9, -9.29)	-24.8 (-29.5, -20.0)
BMI	456	< 0.001	< 0.001	-6.22 (-11.0, -1.46)	-12.5 (-17.2, -7.70)	-26.0 (-30.6, -21.3)
hip	374	< 0.001	< 0.001	-4.29 (-9.33, 0.76)	-8.80 (-14.7, -2.94)	-23.6 (-28.6, -18.5)
Waist	382	< 0.001	< 0.001	-3.21 (-8.91, 2.49)	-9.63 (-14.7, -4.53)	-21.1 (-26.4, -15.7)
Chest	391	< 0.001	< 0.001	-9.64 (-14.3, -4.93)	-13.3 (-18.6, -8.00)	-29.5 (-34.8, -24.2)
WHR	371	0.007	0.01	-5.51 (-11.4, 0.36)	-2.31 (-8.32, 3.71)	-9.81 (-15.8, -3.83)
Parity	588	< 0.001	< 0.001	-5.66 (-11.3, -0.05)	-6.41 (-11.1, -1.75)	-10.8 (-16.4, -5.31)
						
				**Quartile 2**	**Quartile 3**	**Quartile 4**
	**N**	**P_trend_**	**P_het_**	**Coefficient** **(95% CI)**	**Coefficient** **(95% CI)**	**Coefficient** **(95% CI)**
**MRI dense volume (×10^3^, log)**						
age	651	0.24	0.57	-0.05 (-0.18, 0.08)	-0.03 (-0.17, 0.12)	-0.10 (-0.25, 0.04)
weight	515	0.12	0.38	0.07 (-0.09, 0.23)	0.13 (-0.03, 0.29)	0.11 (-0.05, 0.28)
BMI	513	0.75	0.47	0.10 (-0.05, 0.26)	0.10 (-0.06, 0.26)	0.02 (-0.14, 0.19)
hip	425	0.072	0.13	0.12 (-0.04, 0.29)	0.21 (0.02, 0.40)	0.13 (-0.04, 0.31)
Waist	434	0.063	0.19	-0.03 (-0.21, 0.15)	0.12 (-0.04, 0.28)	0.13 (-0.05, 0.31)
Chest	445	0.021	0.034	0.07 (-0.09, 0.22)	0.25 (0.08, 0.42)	0.15 (-0.04, 0.33)
WHR	423	0.25	0.64	0.00 (-0.18, 0.18)	0.08 (-0.10, 0.26)	0.09 (-0.10, 0.27)
Parity	629	0.14	0.46	-0.13 (-0.31, 0.05)	-0.10 (-0.25, 0.04)	-0.11 (-0.29, 0.06)
						
**Cumulus dense volume (×10^3^)**						
age	607	0.35	0.21	-187 (-433, 59.0)	15.1 (-252, 283)	-201 (-464, 61.9)
weight	464	0.003	0.017	9.71 (-267, 286)	-233 (-509, 43.4)	-366 (-641, -92.1)
BMI	462	0.001	0.004	52.7 (-223, 328)	-150 (-426, 127)	-419 (-690, -148)
hip	378	< 0.001	< 0.001	95.6 (-181, 372)	47.4 (-274, 369)	-545 (-824, -266)
Waist	386	0.027	0.14	-12.2 (-322, 298)	-135 (-414, 143)	-323 (-613, -31.9)
Chest	397	0.003	< 0.001	77.1 (-187, 341)	79.5 (-219, 378)	-516 (-817, -216)
WHR	375	0.99	0.55	-83.7 (-387, 219)	122 (-187, 432)	-67.6 (-375, 240)
Parity	596	0.001	0.012	-287 (-601, 26.6)	-285 (-545, -25.1)	-447 (-756, -137)

N = number of women for whom data were available. P_trend _and P_het _are *P*-values for the tests of trend (1 df) and heterogeneity (3 df) respectively between quartiles. Coefficients are for each quartile of the variable relative to the lowest quartile.

**Table 2:** Relationship between measures of breast density and six binary variables

	No. 'Yes'	No. 'No'	**coefficient **(**95% CI**)	*P*-value
**MRI % dense volume (log)**				
Ever smoker	229	304	-0.01 (-0.12, 0.10)	0.82
Post-menopausal	108	418	-0.32 (-0.45, -0.19)	< 0.001
Ever pregnant	466	64	-0.22 (-0.39, -0.06)	0.008
Ever tamoxifen use	28	506	-0.44 (-0.67, -0.20)	< 0.001
Previous OC use	426	106	-0.16 (-0.29, -0.02)	0.023
Ever HRT use	17	97	0.01 (-0.32, 0.33)	0.98
				
**Cumulus %dense area**				
Ever smoker	206	270	0.21 (-3.43, 3.86)	0.91
Post-menopausal	101	372	-8.95 (-13.3, -4.60)	< 0.001
Ever pregnant	424	49	-7.10 (-13.0, -1.18)	0.019
Ever tamoxifen use	30	447	-10.4 (-17.8, -3.07)	0.006
Previous OC use	379	96	0.35 (-4.15, 4.86)	0.88
Ever HRT use	12	73	1.11 (-11.2, 13.4)	0.86
				
**Visually Assessed % dense area**				
Ever smoker	202	268	-1.25 (-5.05, 2.56)	0.52
Post-menopausal	101	366	-10.3 (-14.8, -5.75)	< 0.001
Ever pregnant	418	49	-8.16 (-14.3, -2.03)	0.009
Ever tamoxifen use	29	442	-13.0 (-20.8, -5.28)	0.001
Previous OC use	374	95	-0.39 (-5.08, 4.31)	0.87
Ever HRT use	11	71	5.34 (-7.16, 17.8)	0.41
				
**MRI dense volume (×10^3^, log)**				
Ever smoker	226	303	0.05 (-0.07, 0.16)	0.43
Post-menopausal	107	415	-0.05 (-0.19, 0.09)	0.50
Ever pregnant	463	63	-0.17 (-0.34, 0.00)	0.05
Ever tamoxifen use	28	502	-0.07 (-0.32, 0.18)	0.60
Previous OC use	423	106	-0.08 (-0.22, 0.06)	0.24
Ever HRT use	17	97	0.12 (-0.24, 0.47)	0.52
				
**Cumulus dense area (×10^3^)**				
Ever smoker	206	270	41.9 (-156, 240)	0.68
Post-menopausal	101	372	-251 (-491, -10.6)	0.041
Ever pregnant	424	49	-273 (-596, 49.7)	0.098
Ever tamoxifen use	30	447	-273 (-677, 131)	0.19
Previous OC use	379	96	56.1 (-189, 301)	0.65
Ever HRT use	12	73	180 (-419, 778)	0.56

### Statistical analysis

The primary analyses were based on three measures of proportional density (MRI percent dense volume, Cumulus-measured percent mammographic dense area and visually-assessed percent mammographic percent dense area on a 21-point scale) and two measures of absolute density (MRI dense volume and Cumulus mammographic dense area), as described above. All analyses of mammographic density refer to the MLO projection unless otherwise specified.

The relationships between the density measures for the left and right breasts were assessed using Pearson linear correlation coefficients and t-tests. The mean unsigned difference between breasts (i.e. not taking into account which of a woman's breasts is the denser) is also presented. The relationship between the MRI percent dense volume and Cumulus percent dense area was assessed using linear regression. Correlation coefficients and weighted kappa statistics (assuming equal spacing between the four quartiles derived on the basis of the full dataset) were used to describe inter-reader (MRI) and intra-reader (Cumulus and Visual Assessment (VA)) consistencies.

Linear regression was used to examine associations between the density measures and a range of standard variables, with the two MRI measures log-transformed to closer approximate a normal distribution. Continuous variables were divided into quartiles; age at study registration, weight, body mass index (BMI, defined as weight in kg divided by the square of height in metres), hip, waist and chest circumferences and waist:hip ratio (WHR, defined as the ratio of the waist circumference to the hip circumference). Parity was defined as the number of full-term pregnancies; women with four or more were considered as a single group. The following binary variables were also considered: current/previous smokers *vs *never smokers; women who had ever been pregnant *vs *never pregnant; women who had ever used hormone replacement therapy *vs *those who had not; women who had previously taken an oral contraceptive *vs *those who had not; women who had ever taken tamoxifen as part of a prevention trial *vs *those who had not. Women who reported that their menstrual periods had stopped due either to a natural menopause, a hysterectomy or an oophorectomy were considered as being postmenopausal.

The mean values of each density measure for the *BRCA1*/2 genetic groups were adjusted for age at registration ≥ 45 years and associations between genetic status and density were tested using linear regression. The analyses were repeated additionally adjusting for BMI and parity.

Finally, Poisson regression was used to test whether each of the density measures was associated with cancer risk in this population by quartile of density, and also grouping the two lower and two upper quartiles in order to improve the stability of the estimates. Each individual's at-risk period was defined as starting at their study registration and continuing until the earliest of breast cancer diagnosis, death, prophylactic bilateral mastectomy, other loss to the ONS flagging system or 31 July 2007. The analyses were performed adjusting only for age group, adjusting for genetic status and/or BMI and parity, and also separately for *BRCA1 *mutation carriers, *BRCA2 *mutation carriers and uninformative/non-carriers. The power to detect a doubling of risk between the upper and lower halves of the density distribution was 72% for the MRI percent dense volume and 59% for the Cumulus percent dense area, rising to 92% and 82% for a 2.5-fold increase in risk.

All statistical tests are two-sided and were conducted using Stata version 10.0 (StataCorp, 4905 Lakeway Drive, College Station, Texas 77845, USA).

## Results

It was possible to estimate the MRI breast dense volume for 655 of the women, while suitable mammograms were obtained and read using the Cumulus density-estimation program for 607 women, all but eight of whom were also assessed on the 21-point Visual Assessment (VA) score. For all techniques there was strong concordance between the percentage densities of the left and right breasts with neither side being consistently denser than the other (*P *= 1.0 for MRI, *P *= 0.32 for Cumulus, *P *= 0.31 for VA) [Additional data file 3]. Where both sides were available, the analyses were based on the mean density over both sides [Table S2 in Additional data file 2]; note that no MRI image was recorded as having zero dense volume.

**Figure 1:**
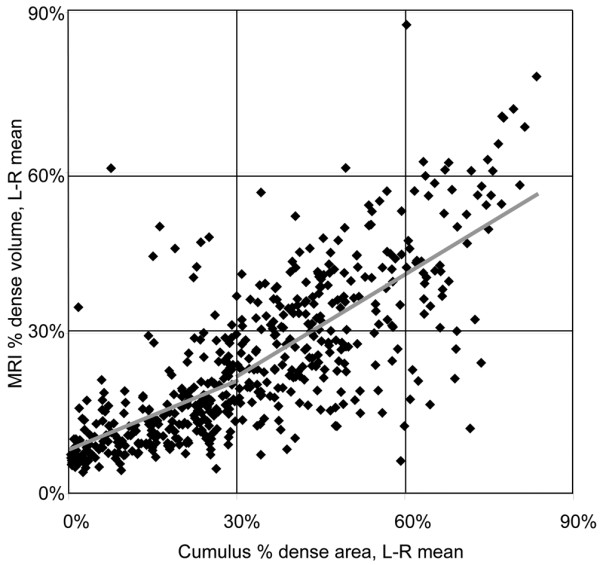
Relationship between MRI percentage dense volume and Cumulus percentage dense area (n = 509). The least-squares best-fit lines for women with cumulus percent dense areas < 30% and ≥ 30% are shown in grey.

The percent dense volume measured by MRI was positively correlated with the percent dense area estimated using the Cumulus program (Figure 1, correlation coefficient = 0.76), but was on average 8.1 percentage points lower (95% CI 7.0 to 9.3, *P *< 0.0001). The relationship between these measures appears to be non-linear. There was a steeper slope for the denser breasts [slope = 0.64 (0.53 to 0.74, *P *< 0.001) for women with a Cumulus dense area ≥ 30%], compared with women with a Cumulus-density < 30% [slope = 0.43 (0.32 to 0.54, *P *< 0.001)] (*P *= 0.0036 for the difference between the two slopes). The 30% cut-off was based on visual inspection of the scatter-plot. On average the MRI percent dense volume was 14.9 percentage points lower (95% CI 13.5 to 16.4) than the Cumulus percent dense area (*P *< 0.0001) for the women with mammographically denser breasts, but there was no significant difference for the less dense breasts (*P *= 0.20). Despite the strong correlation there were a few women for whom the MRI and Cumulus estimates were highly divergent. The correlation between the MRI absolute dense volume and the Cumulus absolute dense area was clearly present but was weaker than for the corresponding percent densities (R = 0.61) [Additional data file 4].

The MRI percent dense volume estimates were highly consistent between the two readers for the 97 images read by both, with a correlation coefficient of 0.94 and a weighted inter-reader kappa of 0.74 based on the quartiles. The Cumulus and VA mammographic percent dense areas were each read by a single reader; 158 images were independently read twice by the Cumulus reader and 93 images were independently read twice by the VA reader. The intra-reader correlation coefficients were 0.90 and 0.92 respectively and weighted kappa coefficients of 0.72 and 0.73 respectively.

The relationships between breast density and a series of standard anthropometric and reproductive/hormone-related factors were assessed for the MRI and mammographic density measures (percent and absolute dense areas and volumes), in order to establish whether the relationships seen in unselected populations are also true in this high-risk group of women, and to see whether breast density as measured by our novel MRI-density estimation technique is related to the same factors as mammographic breast density (Tables 1 and 2).

The Cumulus percent dense area was negatively associated with BMI, weight, hip, waist and chest circumferences and with parity (trend *P *< 0.001 in each case) and with WHR (*P *= 0.003), as expected. Although there was a significant negative trend in Cumulus percent density with increasing age, a significant decrease was seen only for women in the oldest age quartile (≥ 45 years; *P *= 0.001). Post-menopausal women, women who had ever been pregnant and women who had ever used tamoxifen also had lower percent dense areas (*P *< 0.001, *P *= 0.019 and *P *= 0.006 respectively).

Lower MRI percent dense volume was significantly associated with all of the same variables, and additionally showed a possible negative association with previous oral contraceptive use (*P *= 0.023).

The Cumulus measure of absolute dense area was significantly associated with the same variables as the Cumulus percent density, apart from age, tamoxifen use and ever-pregnancy, but the associations were in each case less significant than for percent dense area. The MRI measure of absolute dense volume showed a modestly significant association with chest circumference (*P *= 0.021) but not with any of the other tested variables.

The results of the univariate analyses were used as the starting point for multivariate analyses. For the Cumulus and MRI percent density measures, the best fitting models both consisted of terms for age ≥ 45 years, parity, BMI (trend by quartiles) and chest circumference (trend by quartiles). However, chest circumference was only available for 491 women and so, to avoid excessive reduction in sample sizes, subsequent analyses were conducted under two models: 1) adjusted for age ≥ 45 years only; 2) adjusted for age ≥ 45 years, BMI quartiles and parity.

### Breast density measures and *BRCA1/BRCA2 *genotype

**Figure 2:**
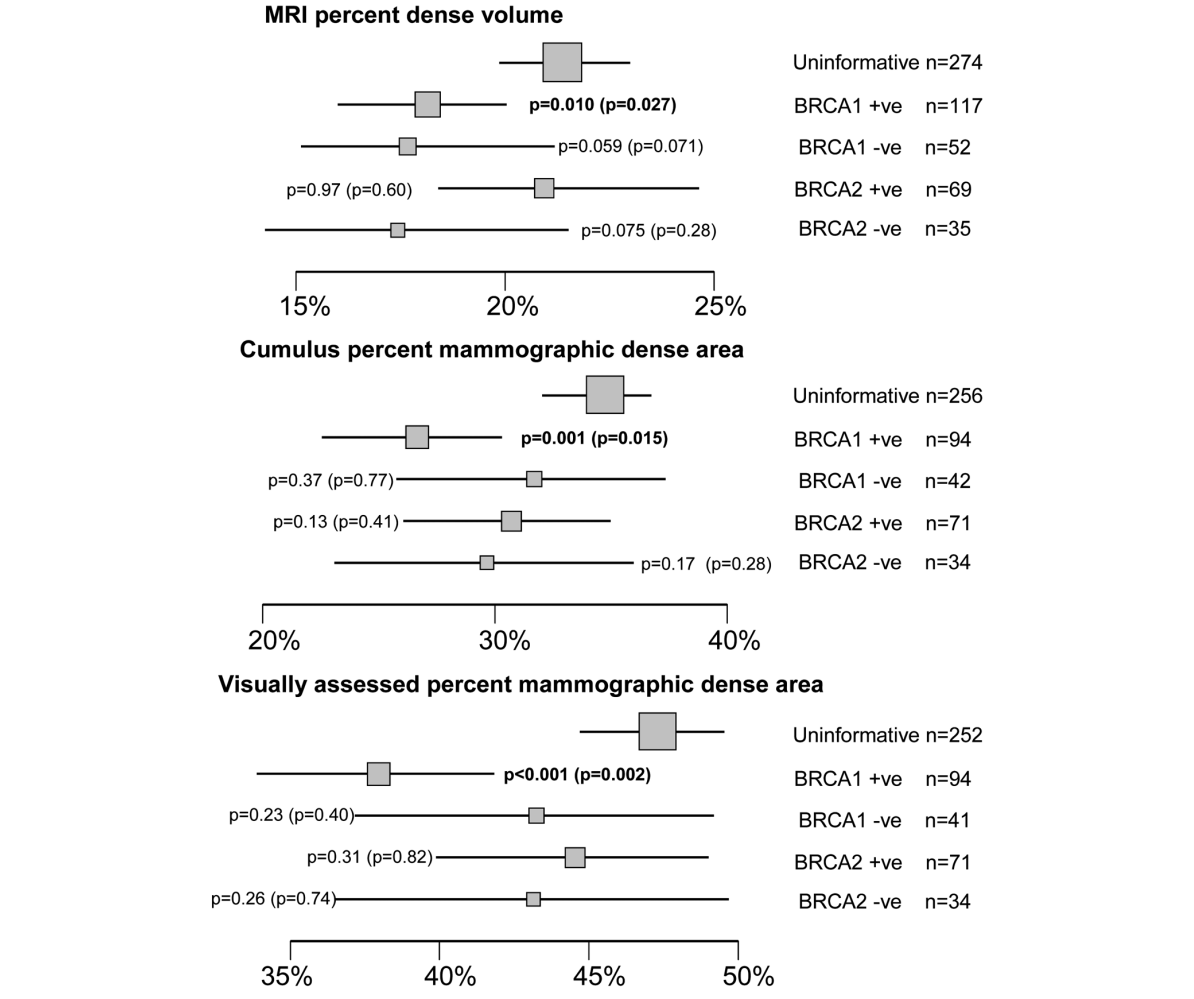
Age-adjusted means and 95% confidence intervals for MRI percent dense volume, Cumulus percent dense area and visual assessment percent dense area by *BRCA1/BRCA2 *genetic status. *P*-values are for the comparisons with the group of women with uninformative *BRCA1*/2 genetic test results, as assessed by linear regression (*P*-values further adjusted for BMI and parity are given in parentheses). Arithmetic means are presented for the Cumulus and Visual measurements; geometric means are shown for the MRI measure (for which the regression analyses were based on log-transformed values). Box areas are proportional to sample size.

**Figure 3:**
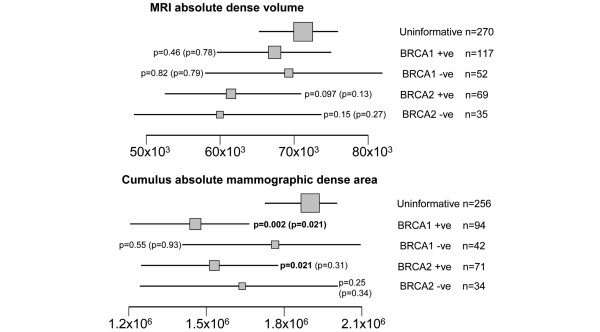
Age-adjusted means and 95% confidence intervals for MRI absolute dense volume and Cumulus absolute dense volume by *BRCA1/BRCA2 *genetic status. *P*-values are for the comparisons with the group of women with uninformative *BRCA1*/2 genetic test results, as assessed by linear regression (*P*-values further adjusted for BMI and parity are given in parentheses). Arithmetic means are presented for the Cumulus measurement; geometric means are shown for the MRI measure (for which the regression analyses were based on log-transformed values). Box areas are proportional to sample size.

Figures 2 and 3 show the means of the age-adjusted percent and absolute breast density measures according to *BRCA1/BRCA2 *genotype. *TP53 *carriers and others with a Li-Fraumeni syndrome family history were not included in this part of the analysis because of their small numbers. For each of the percent and absolute density measures, women with an uninformative genetic test (the baseline group) tended to have denser breasts than either the *BRCA1 *or *BRCA2 *carriers or non-carriers. The comparison with non-carriers was only significant for the MRI percent density, and then only when *BRCA1 *and *BRCA2 *non-carriers were combined (*P *= 0.015 adjusted only for age ≥ 45; *P *= 0.049 adjusted for age ≥ 45, BMI and parity).

*BRCA1 *mutation carriers had significantly lower MRI percent dense volume (*P *= 0.010), Cumulus percent dense area (*P *= 0.001), VA percent dense area (*P *< 0.001) and Cumulus absolute dense volume (*P *= 0.002) than the uninformative group; these effects remained significant after adjustment for BMI and parity (Figures 2 and 3), but their MRI absolute dense volume was no different from that of the uninformative group (*P *= 0.46, Figure 3). *BRCA2 *mutation carriers did not differ from the uninformatives for any density measures except Cumulus absolute dense area (*P *= 0.021), and this result was non-significant after adjustment for BMI and parity (*P *= 0.31).

The data were reanalysed in terms of the Boadicea-estimated *BRCA1 *and *BRCA2 *carrier probabilities (tested carriers and non-carriers were assumed to have probabilities of 1 or 0, as appropriate) using multivariate regression. *BRCA1 *carrier probability was negatively associated with Cumulus percent density (*P *= 0.001, *P *= 0.016 after adjustment for BMI and parity) but not with MRI percent dense volume (*P *= 0.047, *P *= 0.066 adjusted). *BRCA2 *carrier probability was not associated with MRI or Cumulus percent densities (*P *= 0.95 and *P *= 0.24 respectively, age adjusted).

### Breast density measures and risk of breast cancer

Thirty-eight of the 749 women developed breast cancer during the course of the MARIBS study; a further 22 developed breast cancer between their last MARIBS screening round and the cut-off date of 31/07/2007. Unfortunately we were unable to obtain any mammogram for 12 of the women with a breast cancer during the study, so the comparison of the MRI and Cumulus/Visual density measures are not based on equal numbers.

Breast cancer incidence rate showed no significant associations with MRI percent dense volume or Cumulus/Visual percent dense area, except when the analyses were adjusted for *BRCA1 *and *BRCA2 *genotype/carrier probability (Upper two quartiles *vs *lower two quartiles: IRR (incidence rate ratio) = 2.04 (1.16 to 3.59) *P *= 0.013 for MRI; IRR = 2.24 (1.18 to 4.26) *P *= 0.014 for VA). These incidence rate ratios ceased to be significant when further adjusted for parity and BMI, although this may be due to the smaller numbers of women for whom these data were available (Table 3). Stratifying by genotype did not reveal any convincing specific associations between breast cancer incidence and either percent dense area or volume, although the numbers of cancers in each group were small (Table 4). Use of the craniocaudal mammographic view instead of or in addition to the mediolateral oblique view did not appear to improve the predictive value of the Cumulus or Visual percent dense area in this population [Table S3 in Additional data file 2].

**Table 3:** Breast cancer risk according to MRI percent dense volume, Cumulus and VA percent dense area (Medial-Lateral Oblique view)

	No. women	No. cancers	No. pyears	IRR - binary (95% CI) *P*-value	IRR - trend (95% CI) *P*-value
***Adjusted for age ≥ 45 years***					
MRI % dense volume	655	56	4,328	1.68 (0.97, 2.94) 0.066	1.17 (0.92, 1.49) 0.21
Cumulus MLO % dense area	607	42	4,109	1.12 (0.61, 2.05) 0.72	1.06 (0.81, 1.40) 0.66
VA MLO % dense area	599	42	4,060	1.61 (0.87, 2.98) 0.13	1.25 (0.96, 1.64) 0.098
***Adjusted for age ≥ 45 years, tamoxifen use, BMI and parity***					
MRI % dense volume	503	40	3,381	1.32 (0.64, 2.73) 0.46	1.10 (0.79, 1.54) 0.56
Cumulus MLO % dense area	456	30	3,123	0.89 (0.39, 2.03) 0.78	0.92 (0.63, 1.35) 0.68
VA MLO % dense area	450	30	3,085	1.61 (0.71, 3.68) 0.26	1.22 (0.84, 1.76) 0.29
***Adjusted for age ≥ 45 years and BRCA1/2 status/carrier probability***					
MRI % dense volume	619	53	4,083	**2.04 (1.16, 3.59) 0.013**	**1.30 (1.01, 1.67) 0.039**
Cumulus MLO % dense area	591	39	4,006	1.82 (0.96, 3.45) 0.065	1.26 (0.95, 1.68) 0.11
VA MLO % dense area	583	39	3,957	**2.24 (1.18, 4.26) 0.014**	**1.47 (1.11, 1.96) 0.008**
***Adjusted for age ≥ 45 years, BRCA1/2 status/carrier probability, BMI and parity***					
MRI % dense volume	489	39	3,276	1.76 (0.82, 3.77) 0.15	1.26 (0.87, 1.81) 0.22
Cumulus MLO % dense area	450	29	3,078	1.43 (0.62, 3.32) 0.41	1.09 (0.73, 1.63) 0.66
VA MLO % dense area	444	29	3,040	1.84 (0.81, 4.19) 0.15	1.37 (0.94, 2.02) 0.11

*BRCA1*/2 carrier probabilities estimated using Boadicea for untested women and those with an uninformative test result.

Pyears = number of person-years in study and follow-up period

IRR - trend = incidence rate ratio, estimated assuming a linear trend in risk ratio between quartiles

IRR - binary = incidence rate ratio for the higher two quartiles versus the lower two quartiles

CI = confidence interval.

**Table 4:** Breast cancer risk according to MRI percent dense volume and Cumulus and visual assessment percent dense area (Medial-Lateral Oblique view), by *BRCA1/BRCA2 *genetic status

	No. women	No. cancers	No. pyears	IRR (95% CI) *P*-value (adjusted age)	IRR (95% CI) *P*-value (adjusted age, BMI & parity)
***BRCA1 Carriers***					
MRI % dense volume	117	21	687	1.84 (0.77, 4.38) 0.17	1.02 (0.26, 4.05) 0.98
Cumulus MLO % dense area	94	16	579	2.72 (0.99, 7.48) 0.053	1.24 (0.35, 4.37) 0.74
VA MLO % dense area	94	16	579	**4.03 (1.40, 11.6) 0.010**	3.39 (0.94, 12.2) 0.062
***BRCA2 Carriers***					
MRI % dense volume	69	18	385	2.06 (0.78, 5.45) 0.15	3.10 (0.77, 12.4) 0.11
Cumulus MLO % dense area	71	15	418	1.66 (0.60, 4.60) 0.33	**10.4 (1.58, 68.0) 0.015**
VA MLO % dense area	71	15	418	1.75 (0.62, 4.90) 0.29	3.70 (0.64, 21.2) 0.14
***Non-carriers***					
MRI % dense volume	361	14	2,532	**4.49 (1.16, 17.3) 0.030**	2.54 (0.56, 11.6) 0.23
Cumulus MLO % dense area	332	7	2,367	1.13 (0.25, 5.17) 0.87	0.24 (0.04, 1.45) 0.12
VA MLO % dense area	327	7	2,336	1.45 (0.32, 6.66) 0.63	0.43 (0.08, 2.41) 0.34

The *non-carrier *group includes those who tested negative for a known *BRCA1*/2 family mutation as well as those in whom no mutation was

detected after a *BRCA1*/2 mutation screen, because of small numbers.

Pyears = number of person-years in study and follow-up period

IRR = incidence rate ratio for the upper two quartiles relative to the lower two quartiles (the trend test was not attempted due to the small numbers). CI = confidence intervals.

The Cumulus absolute dense area showed no evidence of association with breast cancer incidence (Tables 5 and 6). However, the MRI absolute dense area was associated with an approximately two-fold increase in risk between the higher and lower halves of the distribution, even after adjustment for age ≥ 45, BMI, parity and *BRCA1/BRCA2 *carrier status/probability (IRR = 2.16 (1.12 to 4.17) *P *= 0.021; IRR = 1.35 (1.01 to 1.80) *P *= 0.042 for trend in quartiles) (Table 5). The association appeared to be broadly similar in *BRCA1 *and *BRCA2 *carriers (*BRCA1 *IRR = 3.62 (1.32 to 9.90) *P *= 0.012; *BRCA2 *IRR = 3.06 (1.14 to 8.19) *P *= 0.026) but was not apparent in the non-carriers/uninformative women (*P *= 0.66) (Table 6).

**Table 5:** Breast cancer risk according to MRI absolute dense volume and Cumulus and visual assessment absolute dense areas (Medial-Lateral Oblique view)

	No. women	No. cancers	No. pyears	IRR - binary (95% CI) *P*-value	IRR - trend (95% CI) *P*-value
***Adjusted for age ≥ 45 years***					
MRI dense volume	651	56	4,291	**2.08 (1.20, 3.62) 0.009**	**1.33 (1.05, 1.70) 0.020**
Cumulus MLO dense area	607	42	4,109	0.76 (0.41, 1.41) 0.39	0.96 (0.73, 1.25) 0.75
***Adjusted for age ≥ 45 years, BMI and parity***					
MRI dense volume	499	40	3,345	1.87 (0.98, 3.56) 0.058	1.25 (0.94, 1.66) 0.13
Cumulus MLO dense area	456	30	3,123	0.64 (0.30, 1.36) 0.25	0.84 (0.60, 1.19) 0.33
***Adjusted for age ≥ 45 years and BRCA1/2 status/carrier probability***					
MRI dense volume	615	53	4,046	**2.31 (1.31, 4.07) 0.004**	**1.42 (1.11, 1.83) 0.006**
Cumulus MLO dense area	591	39	4,006	1.19 (0.63, 2.26) 0.59	1.11 (0.82, 1.50) 0.52
***Adjusted for age ≥ 45 years, BRCA1/2 status/carrier probability, BMI and parity***					
MRI dense volume	485	39	3,240	**2.16 (1.12, 4.17) 0.021**	**1.35 (1.01, 1.80) 0.042**
Cumulus MLO dense area	450	29	3,078	0.99 (0.46, 2.13) 0.99	0.95 (0.64, 1.41) 0.81

*BRCA1*/2 carrier probabilities estimated using Boadicea for untested women and those with an uninformative test result.

Pyears = number of person-years in study and follow-up period

IRR - trend = incidence rate ratio, estimated assuming a linear trend in risk ratio between quartiles

IRR - binary = incidence rate ratio for the higher two quartiles versus the lower two quartiles

CI = confidence interval.

**Table 6:** Breast cancer risk according to MRI absolute dense volume and Cumulus and visual assessment absolute dense areas (Medial-Lateral Oblique view), by *BRCA1/BRCA2 *genetic status

	No. women	No. cancers	No. pyears	IRR (95% CI) *P*-value (adjusted age)	IRR (95% CI) *P*-value (adjusted age, BMI & parity)
***BRCA1 Carriers***					
MRI dense volume	117	21	687	**3.62 (1.32, 9.90) 0.012**	3.10 (0.95, 10.1) 0.060
Cumulus MLO dense area	94	16	579	1.54 (0.58, 4.10) 0.39	1.27 (0.39, 4.11) 0.69
***BRCA2 Carriers***					
MRI dense volume	69	18	385	**3.06 (1.14, 8.19) 0.026**	**6.75 (1.80, 25.2) 0.005**
Cumulus MLO dense area	71	15	418	1.03 (0.34, 3.10) 0.96	1.44 (0.38, 5.47) 0.59
***Non-Carriers***					
MRI dense volume	357	14	2,495	1.27 (0.44, 3.65) 0.66	0.79 (0.24, 2.67) 0.71
Cumulus MLO dense area	332	7	2,367	1.14 (0.25, 5.11) 0.87	0.58 (0.11, 3.06) 0.52

The *non-carrier *group includes those who tested negative for a known *BRCA1*/2 family mutation as well as those in whom no mutation was detected after a *BRCA1*/2 mutation screen, because of the small numbers.

Pyears = number of person-years in study and follow-up period

IRR = incidence rate ratio, estimated for the upper two quartiles *vs *the lower two quartiles (the trend test was not attempted due to the small numbers)

CI = confidence interval

## Discussion

We present the results of a large multicentre study designed to explore the value of a novel MRI-based technique for estimating breast density, specifically in a group of women at high genetic risk of breast cancer.

The proportion of the breast volume occupied by dense material, as measured using our MRI-based algorithm, was strongly correlated with the proportion of the mammographic breast area occupied by dense material, as measured by the quantitative Cumulus thresholding algorithm. Previous studies of considerably fewer women have reported broadly similar correlations between various MRI-based and mammography-based density estimates for example [3,10-12]. However, correlation is not the same as equivalence, and it seems that a woman's MRI percent dense volume tends to be lower (on average by eight percentage points) than her Cumulus percent dense area, with a more marked difference among women with denser breasts. The proportional densities estimated by the two methods were notably different for a small number of women; in some cases this may have been due to the use of a later mammogram if an image from the same year as the corresponding MRI was not available, otherwise it may simply reflect the occasional fallibility of human-operated methods.

Mammography separates fatty tissue from tissues of water density on the basis of their differential absorption of the X-ray beam, whereas MRI distinguishes between tissue types on the basis of their behaviours in a magnetic field. Mammographic density estimation is based on a single two-dimensional projection of the breast, in contrast to the three-dimensional MRI image. Furthermore, each method uses a slightly different definition of the extent of the breast, with implications for the degree to which the axillary tail is included. It is hence not surprising that the quantities estimated by the two methods are not the same, despite being closely related. In the absence of a practical, established gold-standard it is not possible to say which is more accurate in any biological sense. However, by examining the relationships between the density estimates and various other factors, including breast cancer risk, we can begin to evaluate their comparative usefulness.

Cumulus percent dense area and MRI percent dense volume were both inversely associated with BMI, weight, hip, waist and chest circumferences, age over 45 years, with postmenopausal status and with tamoxifen use, in line with previous reports specific to Cumulus and other mammographic density methods [13-18]. This confirms firstly that the usual accepted determinants of breast density also apply in this high-risk population, and secondly that they apply similarly to MRI percent dense volume.

We found carrying a *BRCA1 *mutation to be associated with significantly lower mammogram-based density, both in terms of absolute and percentage dense area, but the relationship was less clear for the MRI percent dense area, and was not true for MRI absolute dense volume. There was no evidence of any relationship between *BRCA2 *mutation status and breast density. Mitchell et al 2006 [2] found no relationship between Cumulus percent dense area and *BRCA1 *or *BRCA2 *carrier status in an analysis based on a slightly smaller set of women, but with a higher proportion of mutation carriers and with considerably more women who developed breast cancer (breast cancer cases were not excluded at study recruitment). Specifically, among the set of 176 *BRCA1 *mutation carriers analysed by Mitchell et al, 36% developed breast cancer, compared with only 17% of our 94 *BRCA1 *carriers, hence it is possible that their group of carriers were biased towards women with denser breasts (and thus higher risk, if density modifies breast cancer risk in mutation carriers in the same way that it does in the general population). This would have obscured any more general association between *BRCA1 *mutation status and low density. Conversely, the opposite may well be true, in that we specifically excluded women who had developed breast cancer prior to recruitment (mean age at entry = 40 years). According to the same logic, this could have biased our set of *BRCA1 *carriers towards those with a lower than average density. The same would not necessarily have been seen for *BRCA2 *carriers, in whom a greater proportion would be expected to be asymptomatic at the age of entry regardless of the presence or absence of potential modifiers such as density. The observation of lower density in *BRCA1 *mutation carriers would seem to be at odds with a report that only BRCA1/p53-deficient mice developed lateral branches and alveoli without pregnancy [19]. To our knowledge, there are no other reports that examine this question, although there are several studies reporting textural differences between *BRCA1/2 *carriers and non-carriers from a Chicago study [20-23].

The chief goal of breast density estimation is for the study and prediction of breast cancer risk. High breast density is well established as one of the strongest known risk factors for breast cancer, with clearer associations for quantitative measures of percent density (such as Cumulus) than for qualitative measures [1]. However, this is the first study to evaluate the relationship between MRI-measured density and breast cancer risk. Although the estimated rate ratios for all of the percentage density measures were in the direction of a positive association, none achieved statistical significance after taking into account the multiple testing. This is in contrast with previous findings both in the general population and specifically in *BRCA1*/2 mutation carriers.

Mitchell et al reported a breast cancer odds ratio of 2.29 (95% CI 1.23 to 4.26) for a combined group of *BRCA1*/2 mutation carriers, comparing women with percent density ≥ 50% with those with density < 50%, but found the comparison to be non-significant when densities between 25 to 50% were compared with < 25% [2]. They reported no difference in effect size between *BRCA1 *and *BRCA2 *carriers. Only 17% of the women in our study had a Cumulus percent density ≥ 50% (as opposed to 45% of the women in the Mitchell et al. study), giving us a 30% power to detect a difference of the same magnitude as that reported by Mitchell et al. This difference in the distribution of densities between the studies may be due to differences in ascertainment criteria, as discussed above, or may simply reflect the use of different digitizers.

Our results also appear at first glance to be different from those seen in the general population; a recent meta-analysis of 42 studies reported an almost five-fold increase in risk for the densest category (≥ 75%) versus the least dense (< 5%) [1]. However, the same meta-analysis gave more modest relative risks for less extreme comparisons (e.g. RR = 2.11, 95% CI 1.70 to 2.63 for 25% to 49% versus < 5%, a comparison for which our power was just 14%, based on 21 cancers). Only 10 of our women had a Cumulus percent density ≥ 75%, and our comparison of the upper and lower halves of the distribution was effectively a comparison of those with Cumulus percent densities above or below 29%, and so it is likely that we did not have the statistical power to detect the kind of the effect size that would be realistic for this comparison, whilst the small numbers of cancers precluded any comparison of more extreme levels of density. We did however also test for trends in risk across quartiles of the density distributions, but found no additional significant results. The set of breast cancers comprises a mixture of incident and prevalent cases, but the small numbers do not allow separate analyses of these groups.

The MARIBS study was originally designed to have suitable power to detect differences in sensitivity between MRI and XRM breast screening [5], rather than for testing associations between density and risk. While we were unable to detect any significant associations between percent density and breast cancer risk, it is important to note that the results for the MRI percent dense volume were broadly similar to those for Cumulus percent dense area (despite MRI density being available for more women). Therefore, it seems unlikely that MRI percent density will be a markedly better predictor of cancer risk than the conventional quantitative mammographic density measures, but may prove useful in cases such as *TP53 *mutation carriers for whom x-ray mammography is not considered safe.

We also examined the value of the craniocaudal view in our data set in the light of a recent report that the predictive value of visually-assessed mammographic percent density based on a mediolateral oblique view alone was substantially improved when combined with density estimates from the craniocaudal view [24]. We found no improvement after averaging over both views, although our study may be underpowered to detect such an effect, especially since the craniocaudal image was not available for all of our subjects.

Interestingly, the one density outcome for which we did see a consistent association with breast cancer risk, even after adjusting for genetic status, BMI and parity, was the absolute MRI dense volume, a measure which has not been studied before. There appeared to be an approximate doubling of risk between the upper and lower halves of the distribution, rising to an over three-fold increase in *BRCA1 *and *BRCA2 *carriers. It is possible that this is a false positive result. If it is true, it is curious that MRI dense volume showed almost no significant associations with any of the tested anthropometric or hormonal factors. However, given that breast cancer is believed to be initiated in the stromal or epithelial cells, it is plausible that the total quantity of target tissue is a more relevant predictor of breast cancer risk than the proportional density. In this context it is interesting that the absolute MRI breast volume was the only density measure not found to be lower in BRCA1 mutation carriers. While clearly related to percent dense volume, absolute dense volume is not capturing the same information; we found that 37% of women were in a different half of the percent MRI dense volume distribution than the absolute dense volume distribution, explaining why the results for the two measures were so different. No such breast cancer association was seen for the Cumulus absolute dense area, suggesting that the differences between mammographic and MRI density are more marked when not adjusted for total breast area/volume, and that the three-dimensional MRI dense volume provides a better estimate of the amount of tissue at risk of carcinogenesis. These findings are necessarily preliminary and will need to be confirmed in further studies.

## Conclusions

The MRIBview algorithm for the MRI-based estimation of the volume of dense tissue in the breast provides a viable alternative to quantitative mammographic density estimation and may be of particular value in women at high genetic risk of breast cancer for whom MRI breast screening is already recommended. Although the nature of the available dataset limited our power to detect associations between density and breast cancer risk, MRI percent dense volume did not appear to perform markedly differently from Cumulus percent dense area. However, the association between absolute MRI dense volume and breast cancer risk is a novel, and potentially important finding that requires replication in a specifically designed case-control study.

## Abbreviations

BMI: body mass index; CC: cranio-caudal; CI: confidence interval; IQR: inter quartile range; IRR: incidence rate ratio; MARIBS: magnetic resonance imaging breast screening; MLO: medial-lateral oblique; MLPA: multiple ligation dependant probe amplification; MRI: magnetic resonance imaging; ONS: office of national statistics; RR: relative risk; UCL: University College London; VA: visual assessment; WHR: waist-hip ratio; XRM: X-ray mammography

## Competing interests

RE has received an educational grant from VISTA diagnostics. ML has received salary as a Director of a company (Specialty Scanners) developing dedicated MRI scanners for breast cancer. The company also receives grant funding from the UK Technology Strategy Board. This company does not fund this research or manuscript in any way. ML is employed by the Institute of Cancer Research (University of London) which holds patents on density measurement using MRI and holds the contracts of staff contributing to this research. His department has research agreements with Philips, Siemens and General Electric, major imaging companies that might benefit from this work; these research agreements are not related to the research in this manuscript. ML has also performed paid consultancy for Roche for work unrelated to this manuscript. The other authors declare that they have no competing interests.

## Authors' contributions

DT performed all statistical analyses, prepared the figures and drafted the manuscript. ML, RE, DE and DGE conceived of the study and participated in its design and co-ordination. GK-L coordinated the data management. FL obtained, entered and managed the pedigree data. MK created the MRIBview program. SR and EB performed the MRI density estimates. IW traced and data-managed the mammograms. CB performed the mammographic visual density assessments. SG and SJR performed the BRCA1/2 genetic analyses. RW conceived of the study, participated in its design and coordination, performed the Cumulus density estimation and helped to draft the manuscript. All authors read and approved the final manuscript.

## Supplementary Material

Additional file 2 table S1 (genetic status of participants and probabilities of carrying BRCA1 or BRCA2 mutations for untested women and women with uninformative BRCA1/BRCA2 screening tests as predicted by the BOADICEA program), Table S2 (distributions of the MRI, Cumulus and Visual Assessment measures of breast dense volume and area) and Table S3 (breast cancer risk according to Cumulus and Visual Assessment percent dense area for the craniocaudal view and for the average density over the craniocaudal and medial-lateral oblique views). Click here for file

Additional file 3 Figure S1 (scatter plots showing the relationships between measurements on the left and right breasts for MRI dense volume and percent dense volume, and for Cumulus dense area and percent dense area). Click here for file

Additional file 4 Figure S2 (a scatter plot showing the relationship between MRI absolute dense volume and Cumulus absolute dense area). Click here for file

Additional file 1 The full MARIBS authorship list. Click here for file
