# Supplementary material for: Assessing the usefulness of a novel MRI-based breast density estimation algorithm in a cohort of women at high genetic risk of breast cancer: the UK MARIBS study
Source: Breast Cancer Res. 2009 Nov 11;11(6):R80. doi: 10.1186/bcr2447 (PMC2815542; doi:10.1186/bcr2447)
Supplement: Additional file 2 — table S1 (genetic status of participants and probabilities of carrying BRCA1 or BRCA2 mutations for untested women and women with uninformative BRCA1/BRCA2 screening tests as predicted by the BOADICEA program), Table S2 (distributions of the MRI, Cumulus and Visual Assessment measures of breast dense volume and area) and Table S3 (breast cancer risk according to Cumulus and Visual Assessment percent dense area for the craniocaudal view and for the average density over the craniocaudal and medial-lateral oblique views). [file bcr2447-S2.DOC]

Table S1: BRCA1/BRCA2 genetic status of participants.

| Category | Total no. women | No. women with MRI % dense volume | No. women with Cumulus % dense area | No. women with Boadicea probabilities | Median probability BRCA1 mutation [IQR] | Median probability BRCA2 mutation [IQR] |
| --- | --- | --- | --- | --- | --- | --- |
| Tested BRCA1 mutation carrier | 125 | 117 | 94 |  |  |  |
| Tested BRCA2 mutation carrier | 80 | 69 | 71 |  |  |  |
| Tested BRCA1 mutation non-carrier | 56 | 52 | 42 |  |  |  |
| Tested BRCA2 mutation non-carrier | 41 | 35 | 34 |  |  |  |
| Relative with a BRCA1 mutation; MARIBS woman untested | 27 | 19 | 21 | 27 | 28.2% [24.1%, 30.8%] | 0.070% [0.05%, 0.10%] |
| Relative with a BRCA2 mutation; MARIBS woman untested | 15 | 11 | 15 | 15 | 0.03% [0.01%, 0.07%] | 28.9% [20.0%, 32.1%] |
| Family history of breast or breast/ovarian cancer; no genetic testing | 70 | 45 | 59 | 70 | 3.22% [0.48%, 13.6%] | 2.29% [0.68%, 6.20%] |
| Uninformative BRCA test in MARIBS woman and relative(s) | 9 | 8 | 5 | 8 | 0.68% [0.35%, 7.50%] | 1.05% [0.35%, 4.36%] |
| Uninformative BRCA test in relative(s); no testing in MARIBS woman | 53 | 42 | 43 | 52 | 0.82% [0.18%, 4.41%] | 0.75% [0.35%, 1.51%] |
| Uninformative BRCA test in MARIBS woman; no testing in relatives | 237 | 224 | 208 | 236 | 0.75% [0.13%, 4.18%] | 0.35% [0.11%, 1.34%] |
| TOTAL | 713 | 622 | 592 | 408 |  |  |

The table includes probabilities of carrying BRCA1 or BRCA2 mutations for untested women and women with uninformative BRCA1/BRCA2 screening tests as predicted by the BOADICEA program

The remaining 36 of the 749 women comprised 12 tested TP53 mutation carriers, 5 tested TP53 non-carriers, 4 with uninformative TP53 screening results for themselves and a relative, 3 untested women with a relative with a TP53 mutation and 12 untested women with a family history consistent with Li-Fraumeni syndrome.

Table S2. Distributions of the MRI, Cumulus and VA measures of breast dense volume or area.

|  | MRI % dense volume | Cumulus MLO % dense area | VA MLO % dense area | MRI absolute dense volume (10^3^ pixels) | Cumulus absolute dense area (10^3^ pixels) |
| --- | --- | --- | --- | --- | --- |
| No.women | 655 | 607 | 599 | 651 | 607 |
| Minimum | 2.9% | 0.40% | 2.5% | 2.23 | 27.0 |
| Maximum | 87.7% | 83.4% | 92.5% | 395 | 6394 |
| Median | 21.3% | 29.1% | 45% | 66.5 | 1563 |
| IQR | 13.0% - 33.7% | 15.1% - 45.5% | 30% - 60% | 44.7 – 101 | 842 – 2308 |
| Mean (sd) | 25.0% (15.2%) | 31.5% (20.2%) | 43.8% (20.7%) | 81.3 (55.7) | 1695 (1151) |

The mean density of the two sides was used where both were available.

MLO=medial-lateral oblique, CC=craniocaudal; VA=visually assessed; IQR=inter-quartile range

Table S3: Breast cancer risk according to Cumulus and VA percent dense area for the CC view and the average density over the CC and MLO views.

|  | **No. women** | **No. cancers** | **No. pyears** | **IRR - binary**  **(95% CI) p-value** | **IRR - trend**  **(95% CI) p-value** |
| --- | --- | --- | --- | --- | --- |
| ***Adjusted for age ≥45 years*** | | | | | |
| Cumulus CC % dense area | 481 | 36 | 3,177 | 1.30 (0.67, 2.53) 0.44 | 1.11 (0.83, 1.50) 0.48 |
| VA CC % dense area | 456 | 33 | 3,001 | 1.15 (0.58, 2.29) 0.69 | 1.08 (0.80, 1.46) 0.62 |
| Cumulus CC + MLO % dense area | 481 | 36 | 3,177 | 1.02 (0.53, 1.97) 0.96 | 1.03 (0.77, 1.39) 0.83 |
| VA CC + MLO % dense area | 453 | 33 | 2,984 | 1.42 (0.71, 2.84) 0.33 | 1.27 (0.93, 1.74) 0.13 |
| ***Adjusted for age ≥45 years, tamoxifen use, BMI and parity*** | | | | | |
| Cumulus CC % dense area | 360 | 24 | 2,412 | 1.29 (0.51, 3.29) 0.59 | 1.05 (0.69, 1.60) 0.81 |
| VA CC % dense area | 341 | 21 | 2,280 | 1.22 (0.47, 3.15) 0.68 | 1.13 (0.74, 1.73) 0.58 |
| Cumulus CC + MLO % dense area | 360 | 24 | 2,412 | 0.69 (0.28, 1.70) 0.42 | 0.95 (0.63, 1.45) 0.82 |
| VA CC + MLO % dense area | 338 | 21 | 2,263 | 1.48 (0.57, 3.90) 0.42 | 1.35 (0.86, 2.12) 0.20 |
| ***Adjusted for age ≥45 years and BRCA1/2 status/carrier probability*** | | | | | |
| Cumulus CC % dense area | 470 | 33 | 3,107 | 1.92 (0.96, 3.88) 0.067 | **1.39 (1.01, 1.92) 0.041** |
| VA CC % dense area | 446 | 30 | 2,941 | **2.09 (1.01, 4.30) 0.047** | 1.37 (0.99, 1.88) 0.058 |
| Cumulus CC + MLO % dense area | 470 | 33 | 3,107 | 1.75 (0.87, 3.52) 0.12 | 1.31 (0.95, 1.80) 0.10 |
| VA CC + MLO % dense area | 443 | 30 | 2,924 | 1.96 (0.94, 4.09) 0.073 | **1.58 (1.11, 2.25) 0.011** |
| ***Adjusted for age ≥45 years, BRCA1/2 status/carrier probability, BMI and parity*** | | | | | |
| Cumulus CC % dense area | 356 | 23 | 2,382 | 1.44 (0.55, 3.78) 0.45 | 1.22 (0.78, 1.89) 0.38 |
| VA CC % dense area | 338 | 20 | 2,258 | 1.81 (0.71, 4.61) 0.21 | 1.33 (0.86, 2.04) 0.20 |
| Cumulus CC + MLO % dense area | 356 | 23 | 2,382 | 1.00 (0.39, 2.56) 1.00 | 1.14 (0.73, 1.79) 0.56 |
| VA CC + MLO % dense area | 335 | 20 | 2,241 | 1.54 (0.60, 3.98) 0.37 | 1.53 (0.95, 2.48) 0.080 |

*BRCA1*/2 carrier probabilities estimated using Boadicea for untested women and those with an uninformative test result.

Pyears=number of person-years in study and follow-up period

IRR – trend = incidence rate ratio, estimated assuming a linear trend in risk ratio between quartiles

IRR – binary = incidence rate ratio for the higher two quartiles versus the lower two quartiles
