# Supplementary material for: Assessing the usefulness of a novel MRI-based breast density estimation algorithm in a cohort of women at high genetic risk of breast cancer: the UK MARIBS study
Source: Breast Cancer Res. 2009 Nov 11;11(6):R80. doi: 10.1186/bcr2447 (PMC2815542; doi:10.1186/bcr2447)
Supplement: Additional file 3 — Figure S1 (scatter plots showing the relationships between measurements on the left and right breasts for MRI dense volume and percent dense volume, and for Cumulus dense area and percent dense area). [file bcr2447-S3.DOC]

**Figure S1**

Scatter plots showing the relationships between measurements on the left and right breasts for MRI dense volume and percent dense volume, and for Cumulus dense area and percent dense area.

Mean (sd) values given for each side.

N = number of women for whom measurements on both sides were available.

p=significance of the t-test for a difference in means between sides.

R=correlation coefficient between sides.
