# Supplementary material for: Assessing the usefulness of a novel MRI-based breast density estimation algorithm in a cohort of women at high genetic risk of breast cancer: the UK MARIBS study
Source: Breast Cancer Res. 2009 Nov 11;11(6):R80. doi: 10.1186/bcr2447 (PMC2815542; doi:10.1186/bcr2447)
Supplement: Additional file 4 — Figure S2 (a scatter plot showing the relationship between MRI absolute dense volume and Cumulus absolute dense area). [file bcr2447-S4.DOC]

**Figure S2**

**Relationship between MRI absolute dense volume and Cumulus absolute dense area**

Correlation coefficient = 0.61. Grey lines indicate the medians of each variable’s distribution (66.5x10^3^ pixels for MRI; 1562.8x10^3^ pixels for Cumulus).
