# Supplementary material for: Assessing the usefulness of a novel MRI-based breast density estimation algorithm in a cohort of women at high genetic risk of breast cancer: the UK MARIBS study
Source: Breast Cancer Res. 2009 Nov 11;11(6):R80. doi: 10.1186/bcr2447 (PMC2815542; doi:10.1186/bcr2447)
Supplement: Additional file 1 — The full MARIBS authorship list. [file bcr2447-S1.DOC]

**Appendix (on-line)**

# Study Advisory Group (past and present)

M.O. Leach (Chairman and Principal Investigator)

J. Brown (Health Economist)

A. Coulthard (Consultant Radiologist)

A.K. Dixon (Professor and Honorary Consultant Radiologist)

J.M. Dixon (Consultant Surgeon & Senior Lecturer)

D. Easton (Professor of Genetic Epidemiology)

R.A. Eeles (Reader in Clinical Cancer Genetics and Honorary Consultant in Cancer Genetics & Clinical Oncology)

D.G. Evans (Consultant Geneticist)

F.J. Gilbert (Professor of Radiology)

J. Hawnaur (Consultant Radiologist)

P. Kessar (Consultant Radiologist)

S.R. Lakhani (Professor of Breast Cancer Pathology)

S. Moss (Epidemiologist)

A.R. Padhani (Consultant Radiologist)

A.J. Potterton (Consultant Radiologist)

B.A. J. Ponder (Professor and Head of Department of Oncology)

J. Sloane *(deceased)* (Professor of Pathology)

L.W. Turnbull (Professor of Radiology and Honorary Consultant)

L.G. Walker (Professor of Cancer Rehabilitation)

R.M.L. Warren (Consultant Radiologist)

# Study Staff (past and present)

L.J. Pointon (Study Co-ordinator)

R.J.C. Hoff (Assistant Study Co-ordinator)

K. Chan (Data Manager)

M. Khazen (Image Analysis Physicist)

E. Charles-Edwards (Clinical Physicist)

R.M.L. Warren (Study Radiologist)

J. Anderson (Health Psychologist)

C. Levesley (Psychology Research Assistant)

I. Griebsch (Health Economist)

D. Thompson (Statistician)

C. Hayes (Study Physicist)

R. Gregory (Study Physicist)

G. Charles-Edwards (MR Physicist)

M. Sydenham (Acting Study Co-ordinator)

K. Bletcher (Data Manager)

G.P. Liney (Study Physicist)

B. Browne (Data Manager)

G. Kwan-Lim (Study Co-ordinator)

F. Lennard (Research Assistant)

I. Warsi (Research Manager)

# Data Monitoring and Ethics Committee

K. McPherson (Chairman) (Visiting Professor of Public Health Epidemiology)

R. Blamey (Prof. Emeritus and Cons. Breast Surgeon)

S. W. Duffy (Professor of Cancer Screening)

# Trial Steering Committee

A. Howell (Chairman) Professor of Medical Oncology

D. Easton (Study Statistician) Genetic Epidemiologist

D.G. Evans (Study Representative) Consultant Geneticist

J. E. Husband (Host Institution Representative) Professor of Radiology

E. Maher (Independent Member) Professor of Medical Genetics

M.J. Michell (Independent Member) Consultant Radiologist

R.M.L. Warren (Study Radiologist) Consultant Radiologist

W. Watson (Consumer Representative) Founder of the Hereditary Breast Cancer Group

# Recruiting centres (No. women recruited)

Aberdeen: N.E. Haites, B. Gibbons, H. Gregory, M. McJannett, L. McLennan (29)

Belfast: P.J. Morrison, L. Jeffers (12)

Birmingham: T. Cole, L. Burgess, C. McKeown, J.E.V. Morton (24)

Bristol Royal Infirmary: Z. Rayter (3)

Cambridge: J. Mackay, J Rankin, L.G. Bobrow, S. Downing, S. Everest, A. Middleton, B. Newcombe (67)

Dundee: D. Goudie, D. Young (24)

Edinburgh: M. Steel, E.D.C. Anderson, J. Campbell, J.M. Dixon, P. Walsh (60)

Frenchay Hospital, Bristol: S.J. Cawthorn, M. Shere, C. Dawe (29)

Glasgow: R. Davidson, C.M. Watt (20)

Guy's & St Thomas': S.V. Hodgson, S. Watts (43)

Leeds: C. Chu, G. Turner, E. Hazell, L. Rae (55)

Liverpool: I. Ellis, J. Birch, C. Holcombe, S. Holcombe, K. Makinson (16)

Manchester Regional Genetics Service: D.G. Evans, G. Hall, A. Shenton (157)

Newcastle: F. Douglas, G. Seymour (111)

Northwick Park: J. Paterson, C. Cummings, L. Jackson (9)

Sheffield: O.W.J. Quarrell, J.A. Cook, D. Kumar (14)

Southampton: D.M. Eccles, G. Crawford, S. Goodman (34)

Royal Marsden NHS Foundation Trust, London & Sutton & St George’s (or collaborators who referred to this centre): R.A. Eeles, S. Allen, A. Ardern-Jones, E. Bancroft, C. Brewer, G. Brown, C. Chapman, D.L. Christensen, R.C. Coombes, I. Fentiman, S. Furnell, S. Goff, S. Gray, G. Gui, T. Homfray, R. Houlston, M.W. Kissin, I. Laidlaw, F. Lennard, I. Locke, A.M. Lucassen, K. McReynolds, G. Mitchell, M.W.E. Morgan, U. Querci della Rovere, N. Rahman, S. Shanley, S. Shrotria, N. Sodha, A. Stacey-Clear, C. Webster (130)

# MR Readers (no. cases read)

Aberdeen: F.J. Gilbert (132), G. Needham (75)

Barnet: G.R. Kaplan (19)

Belfast: J.G. Crothers (13)

Birmingham: C.P. Walker (48)

Bristol Royal Infirmary: A. Jones (10)

Cambridge: P.D. Britton (161), A.K. Dixon (104), R. Sinnatamby (25), R.M.L. Warren (759)

Dundee: J.M. Rehman (14), D. Sheppard (20)

Edinburgh: J. Walsh (426)

Frenchay Hospital, Bristol: I.D. Lyburn (23), N.F. Slack (50)

Glasgow: L.M. Wilkinson (24)

Guy's & St Thomas': S. Rankin (222)

Hillingdon Hospital, Middlesex: K. Raza (100)

Hull: G. Hall (81), P. Balan (47), L. Turnbull (221)

Liverpool: G.H. Whitehouse (47)

Manchester - Christie Hospital / Nightingale Centre: C.R.M. Boggis (80), E. Hurley (16), A. Jain (4), S. Reaney (49), M. Wilson (63)

Manchester Medical School: J.M. Hawnaur (183), J. Jenkins (4)

Newcastle: A. Coulthard (234), A.J. Potterton (321)

Northwick Park: B. Shah (57), W. Teh (92)

Paul Strickland Scanner Centre, NW London: A.R. Padhani (269)

Royal Hospital Haslar, Gosport: P.J. Buxton (2), J.M. Domsan (2), P.A.L. Gordon (6)

Southampton: M. Briley (55), C. Rubin (72)

Sutton & St George’s: P. Kessar (now at Bromley Hospitals NHS Trust) (256)

UCH: M.A. Hall-Craggs (23)

# XRM Readers (no. films read)

Aberdeen: H.E. Deans (42), K. Duncan (47), L. Gomersall (30), G. Iyengar (3), G. Needham (8)

Barnet: G.R. Kaplan (4)

Belfast: J.G. Crothers (12), J. McAllister (12), J.M. Kirby (1)

Birmingham: S. Bradley (47), M.G. Wallis (45)

Bristol Royal Infirmary: J.E. Basten (56), E. Kutt (52)

Cambridge: P.D. Britton (185), R. Davies (5), C.D.R. Flower (9), A.H. Freeman (240), D. O'Driscoll (4), R. Sinnatamby (310), R.M.L. Warren (426)

Dundee: A.M. Cook (25), C.M. Walker (25)

Edinburgh: A. Buttimer (55), A. Gilchrist (35), B.B. Muir (106), J. Murray (126), L. Smart (10), M. Smith (8)

Glasgow: C. Cordiner (18), J. Litherland (16)

Guy's & St Thomas': A. Jones (51), S. McWilliams (76)

Hull: A.E. Hubbard (146)

Liverpool: A. Ap-Thomas (1), D.A. Ritchie (33), F. White (32)

Manchester - Christie Hospital / Nightingale Centre: D.L. Asbury (46), U. Beetles (14), C.R.M Boggis (212), R. Dobrashian (3), M.D.J. Harake (15), E. Hurley (34), A. Jain (20), S. Reaney (74), M. Wilson (117)

Newcastle: B. Kaye (55), M. McElroy (180), L. McLean (145), W. Wotherspoon (230)

Northwick Park: G. Markham (8)

Southampton: A. Bisset (2), S. Hegarty (57), G. Michaels (59), N. Robson (2)

Sutton & St George’s: J. Husband (6), K.T Khaw (1), D. MacVicar (10), E. Moskovic (7), J. Murfitt (23)

# Other radiology/MR staff

Aberdeen: M.L. Muirhead, T.W. Redpath, S. Semple

Barnet: M. Cunningham, S. Turnell

Belfast: C. Reynolds

Birmingham: P. Fergusson, Z. Vegnuti

Bristol Royal Infirmary: S. Cowley, K. Isaacs, P. Richardson

Cambridge: J. Green, J. Pinney, C. Pittock

Dundee: S.J. Gandy, P. Martin, T. McLeay

Edinburgh: T. Lawton, I. Marshall, L. Thomson

Frenchay Hospital, Bristol: H. Albarran, V. Blake, J. Robson

Glasgow: M. Cockburn

Guy's & St Thomas': J. Goodey, R. Lund, K. McBride

Hull: S. Hunter, G. Liney

Liverpool: J. Chance, J. Davies, Z. Hussain

Manchester - Christie Hospital / Nightingale Centre: N. Brown, C. Hammond, W. Johnson

Manchester Medical School: J.E. Adams, Y. Watson

Newcastle: P. English, L. Lewis, M. Myers

Northwick Park: D. Fox, J. Johnson, J. Shah

Paul Strickland Scanner Centre, NW London: L. Culver, R. Sale, J.J. Stirling, N.J. Taylor

Royal Hospital Haslar, Gosport: E. Boyd, J. Evans, W. Johnston, S. Lindsay, R. MacKenzie, B. Tailor, L. Watts

Southampton: A. Darekar, S. King, N. Shepherd

Sutton & St George’s: G. Charles-Edwards, E. Charles-Edwards, E. Scurr (on behalf of all the MRI radiographers, Sutton)
